# Strained achilles tendon complicated by nocturnal dyspepsia probably caused by ibuprofen: a case report

**DOI:** 10.1186/1757-1626-1-31

**Published:** 2008-07-14

**Authors:** Richard Smith

**Affiliations:** 1 Editor-in-Chief, Cases Journal, BioMed Central, Middlesex House, 34-42 Cleveland Street, London, W1T 4LB, UK

## Abstract

A 56-year-old male who is overweight experienced pain in his Achilles tendon after running four miles. He subsequently developed a severe limp. The condition was complicated by nocturnal dyspepsia probably caused by ibuprofen. His pain and limp were gone in six days.

## Case presentation

When staying in hotels I often try to take a run in the morning – because, otherwise, I find that I often get no exercise. At the end of March 2008 I was visiting the National Heart Lung and Blood Institute at Bethesda, a suburb of Washington. On the morning after my arrival I ran about a mile and a half through backstreets. I felt good.

Forty years ago, when I was 16 and at school, I was a moderately successful cross country runner. I several times won our school race, and one year I was the South London Grammar School's intermediate champion. I ran cross-country at university but was much less successful – partly because the competition was more intense and partly because I never trained. In my late 20s I ran occasionally for a club but ceased running competitively by the time I was 30, although I did run the Bath half marathon – in 1 hour 45 minutes – about 15 years ago, when I was in my early 40s. Since then I've run a few miles a couple of months but have continued to cycle regularly – about 25 miles a week.

My second morning in Bethesda I took the advice of a colleague and set off to run round the perimeter of the campus of the National Institutes of Health. This turned out to be rather farther than I expected – about four miles – and to be fairly hilly and to be mostly on road. I didn't run uninterruptedly but stopped a few times. It took me about 35 minutes to complete the run, and I was wearing trainers.

At the end of the run I showered and felt fine. I had a light breakfast, worked in my room, and then took a taxi to Dulles Airport, where I caught a plane to San Diego, a five-hour flight, which I flew in a cramped seat in economy class with somebody beside me. From San Diego Airport I took a taxi about 20 miles to the Northern end of San Diego, where I stayed in a hotel so upscale that I felt intimidated. I arrived in the hotel just after 7 San Diego time, took a stroll of about half a mile with some mild discomfort in my left Achilles heel, ate dinner in my room with a half bottle of wine, and went to bed.

During the night I was woken several times by growing discomfort in my left Achilles heel. I tossed and turned in bed and found it difficult to get comfortable. I did, however, keep returning to sleep.

When I woke in the morning and got out of bed I had considerable discomfort. Indeed, I walked with a very pronounced limp. I hobbled, no other word for it, to breakfast. Walking down the stairs was especially difficult. I contemplated going backwards. My discomfort and Long John Silver limp lasted all day, making me feel moderately ridiculous. I didn't walk any great distance, and the limp was more pronounced than the discomfort.

I didn't take any painkillers during the day, but as it came time to bed I decided I ought to. I'd finished the aspirin I usually carry with me, so I went to the hotel shop. It had only ibuprofen, which I have rarely, if ever, taken before. I took one pill before going to bed – after my dinner, which did include probably three glasses of wine.

During the night I woke with pronounced dyspepsia – which I rarely, if ever, get. I wondered what might have caused it, and after running though a sleepy differential diagnosis I decided it was probably the ibuprofen. I was probably right, as the dyspepsia had gone by the morning. I didn't take anymore ibuprofen and it didn't reappear.

My discomfort and limp were both considerably better on the second day, and by the fourth day I'd stopped limping. I didn't go for another run and flew back to London on the sixth day with only a twinge in my Achilles tendon.

This is now two months ago, and I've been for several runs (but of not more than two miles) and walked the Dalesway with a venture to the highest of the Howgills in the middle (90 miles with a 22 mile walk one day). I've had no recurrence of my problem, but I'm still conscious of twinges. I fear that if I try another longish run it might recur. One day I'll experiment and report back.

## Consent

Written informed consent was not sought for publication of this case report since the author is the subject.

## Competing interests

I am the Editor-in-Chief of *Cases Journal*. I declare no other competing interests.

